# Supplementary material for: Activated carbon ameliorates type 2 diabetes via metabolic remodeling of the gut microbiota
Source: Microbiol Spectr. 2025 Sep 19;13(11):e03109-24. doi: 10.1128/spectrum.03109-24 (PMC12584629; doi:10.1128/spectrum.03109-24)
Supplement: Supplemental material — Fig. S1 to S8; Table S1. [file spectrum.03109-24-s0002.docx]

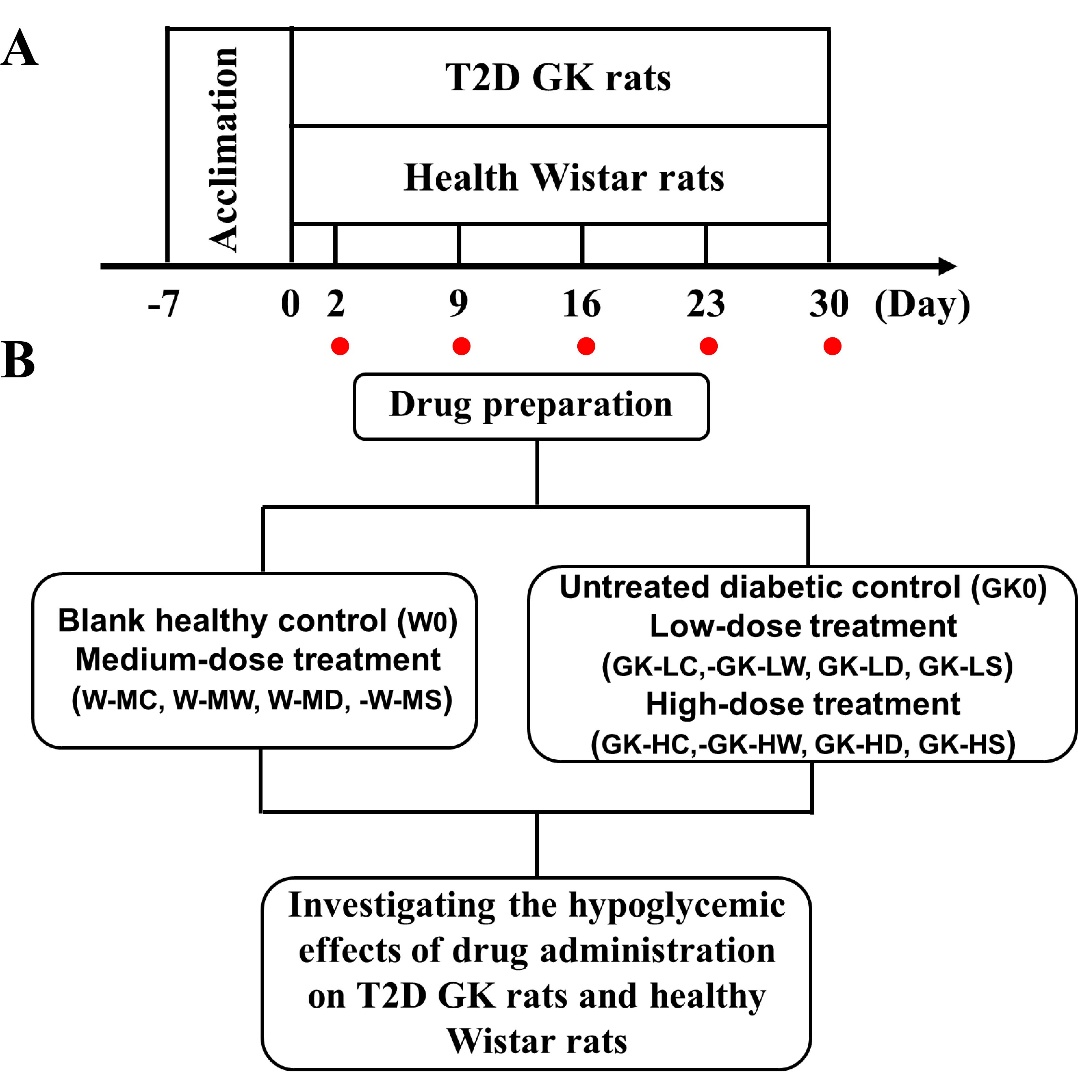


Figure S1 Trial profile. (A) Study design. The red dots indicate the time of each sampling. (B) Participant Flow.


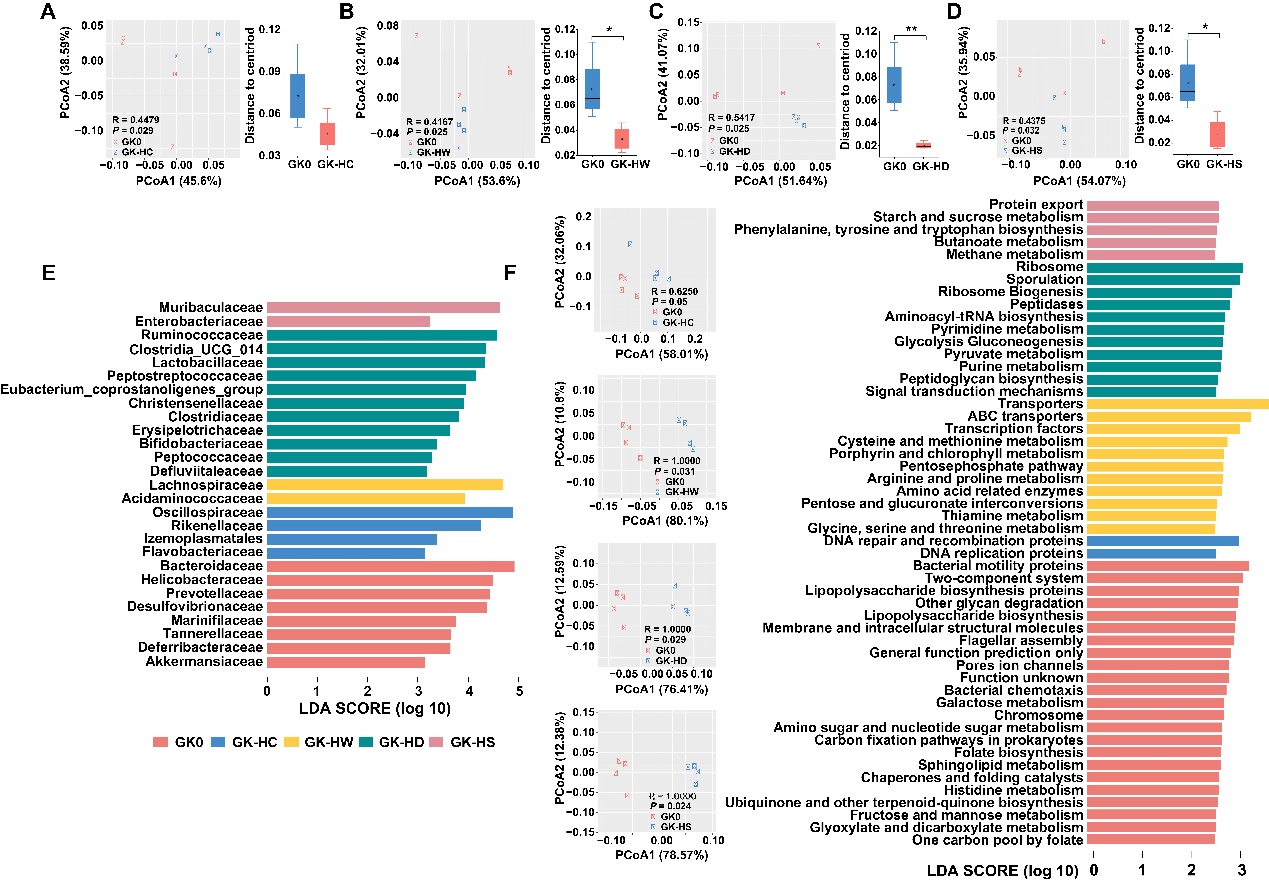


Figure S2 Microbial diversity and functional analysis reveal alterations in gut microbiota and functions in GK groups under high dose dietary drug intervention. (A), (B), (C), (D) Principal coordinate analysis (PCoA) based on weighted-UniFrac distance and inner-group dissimilarity by ANOVA. (E) Differences in bacterial taxonomy at the family level were ranked according to the LefSe analysis. (F) Variation in predicted metabolic capacity among gut communities. The PCoA plot based on the Bray-Curtis distance of KOs are shown on the left, while the differences in predicted metabolic functions are shown on the right (*P* < 0.05 and LDA > 2.5).


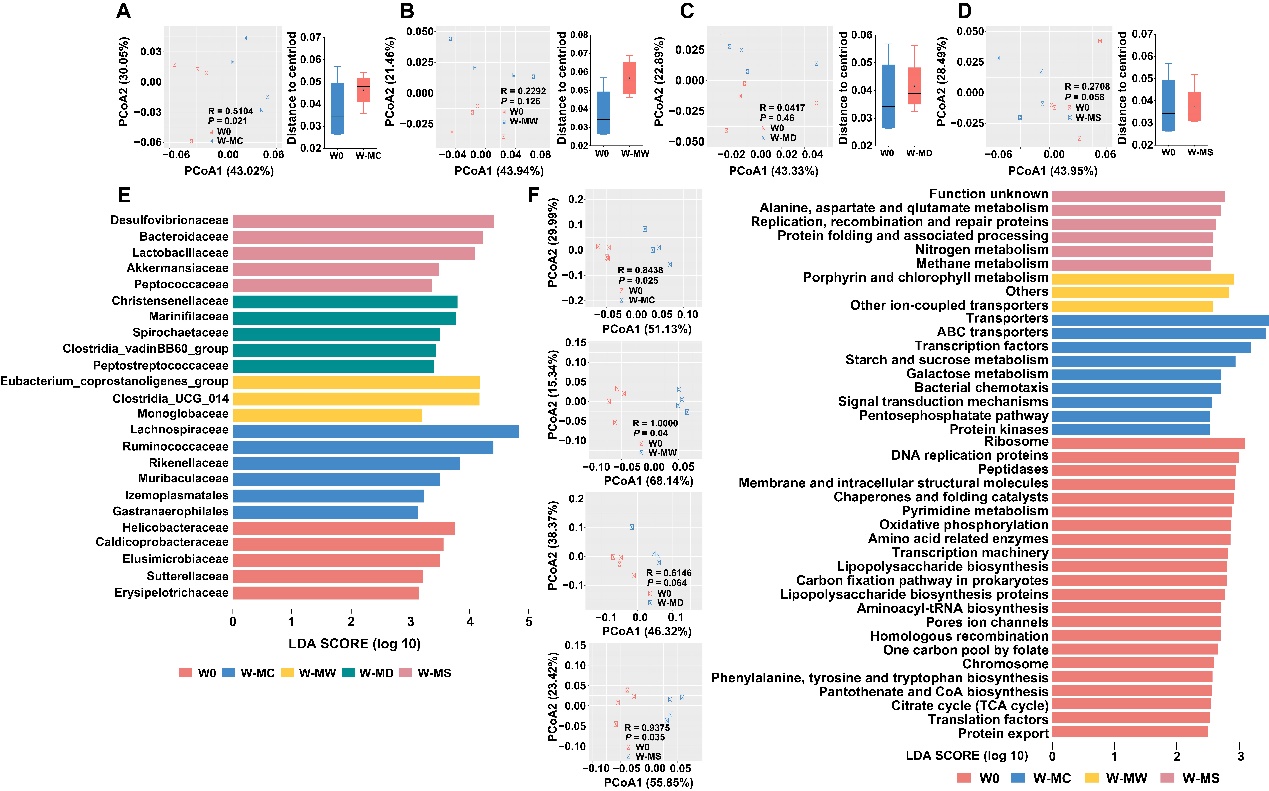


Figure S3 Microbial diversity and functional analysis reveal alterations in gut microbiota and functions in Wistar groups under medium dose dietary drug intervention. (A), (B), (C), (D) Principal coordinate analysis (PCoA) based on weighted-UniFrac distance and inner-group dissimilarity. (E) Differences in bacterial taxonomy at the family level were ranked according to the LefSe analysis. (F) Variation in predicted metabolic capacity among gut communities. The PCoA plot based on the Bray-Curtis distance of KOs by ANOVA are shown on the left, while the differences in predicted metabolic functions are shown on the right (*P* < 0.05 and LDA > 2.5).


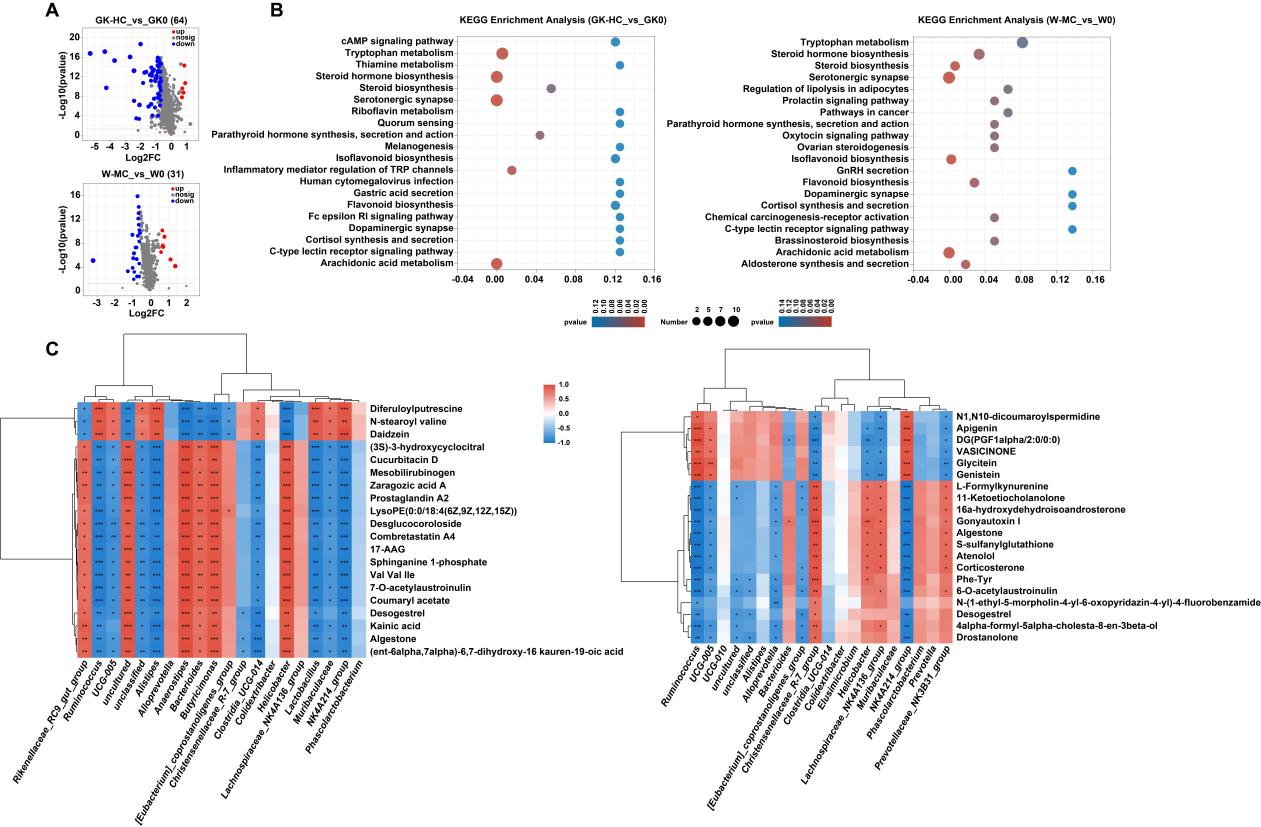


Figure S4 Fecal metabolic patterns in the medium (W1) and high (GK5) dose activated carbon treated groups. (A) Volcano maps of differential metabolites. Significantly upregulated and downregulated genes are shown in red and blue, respectively. Genes with no significant changes (nosig) in transcription are shown in grey. (B) KEGG pathway enrichment analysis. (C) Heatmap analysis of the Pearson correlation in GK5 (left) and W1 (right) groups. The red squares indicate positive correlations, whereas the blue squares indicate negative correlations. The metabolite clustering tree is shown on the left. The distance between branches shows the closeness in the expression pattern of metabolites.


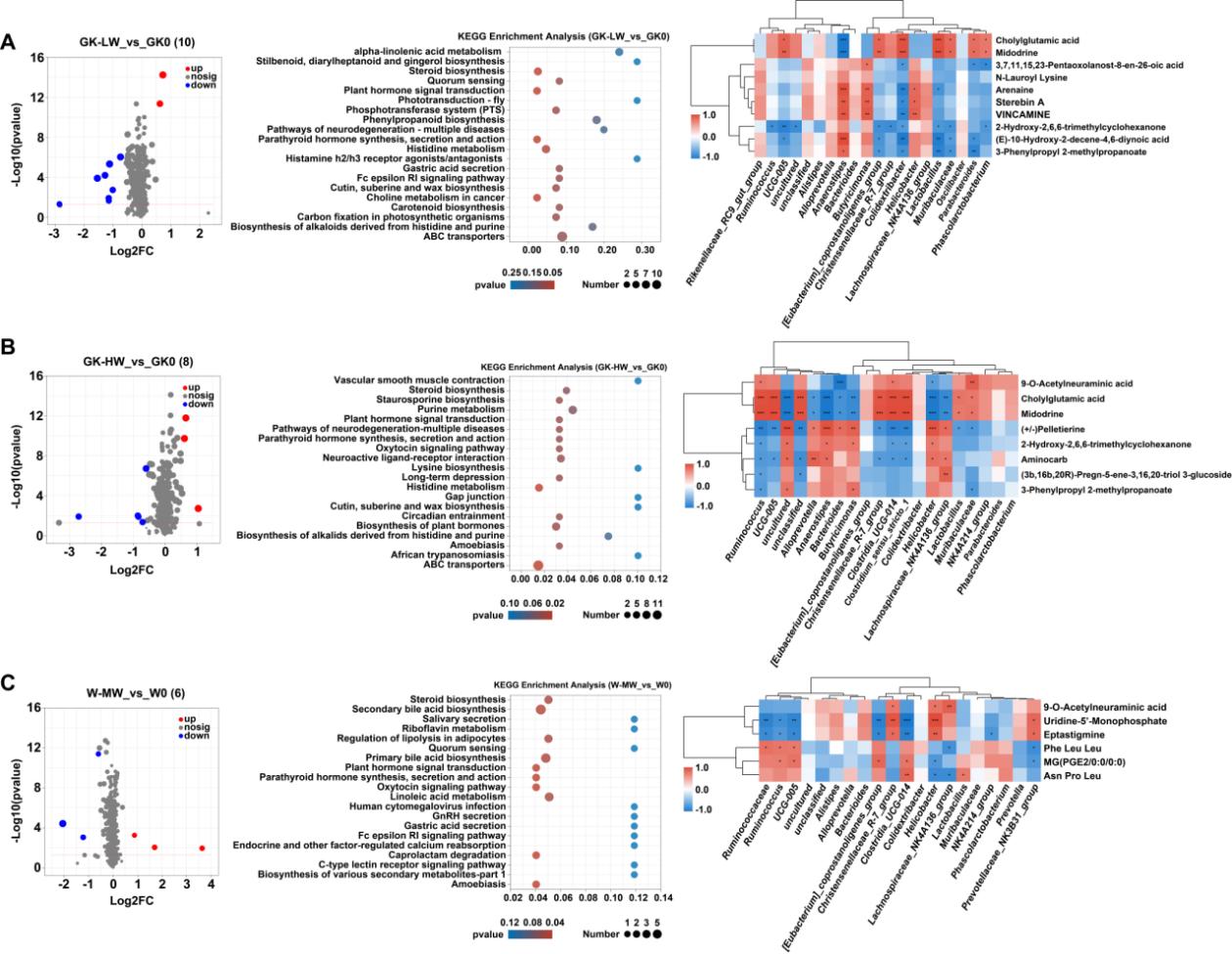


Figure S5 Fecal metabolic patterns in the low (GK2) (A), high (GK6) (B) and medium (W2) (C) dose wheatgrass treated groups. The Volcano maps of differential metabolites are on the left. Significantly upregulated and downregulated genes are shown in red and blue, respectively. Genes with no significant changes (nosig) in transcription are shown in grey. KEGG pathway enrichment analysis are in the middle. Heatmap analysis of the Pearson correlation are on the right. The red squares indicate positive correlations, whereas the blue squares indicate negative correlations. The metabolite clustering tree is shown on the left. The distance between branches shows the closeness in the expression pattern of metabolites.


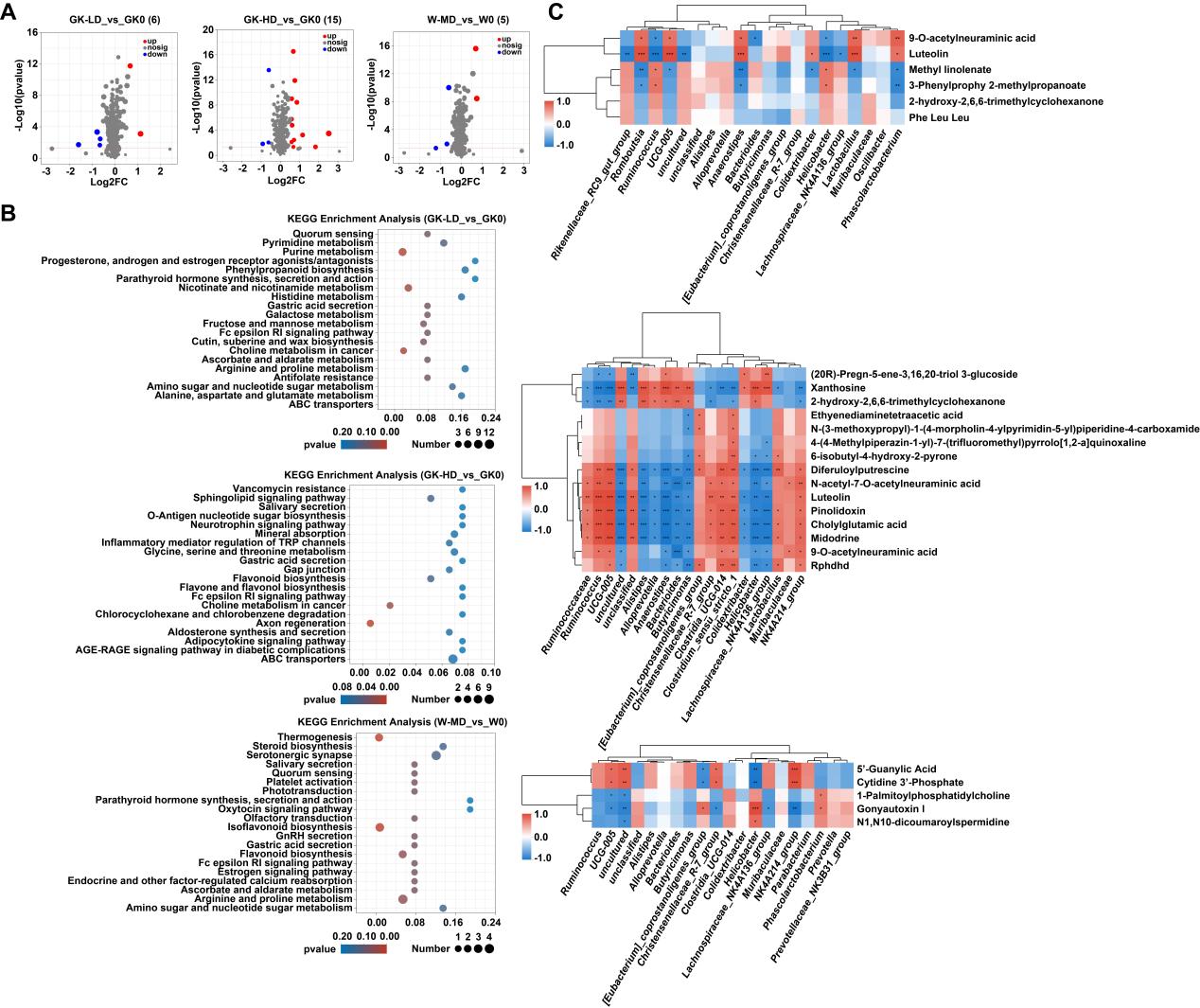


Figure S6 Fecal metabolic patterns in the low (GK3), high (GK7) and medium (W3) dose dandelion treated groups. (A) Volcano maps of differential metabolites. Significantly upregulated and downregulated genes are shown in red and blue, respectively. Genes with no significant changes (nosig) in transcription are shown in grey. (B) KEGG pathway enrichment analysis. (C) The top is the Pearson correlation of GK3 group, the middle heatmap analysis represent the GK7 group and the bottom represent the W3 group. The red squares indicate positive correlations, whereas the blue squares indicate negative correlations. The metabolite clustering tree is shown on the left. The distance between branches shows the closeness in the expression pattern of metabolites.


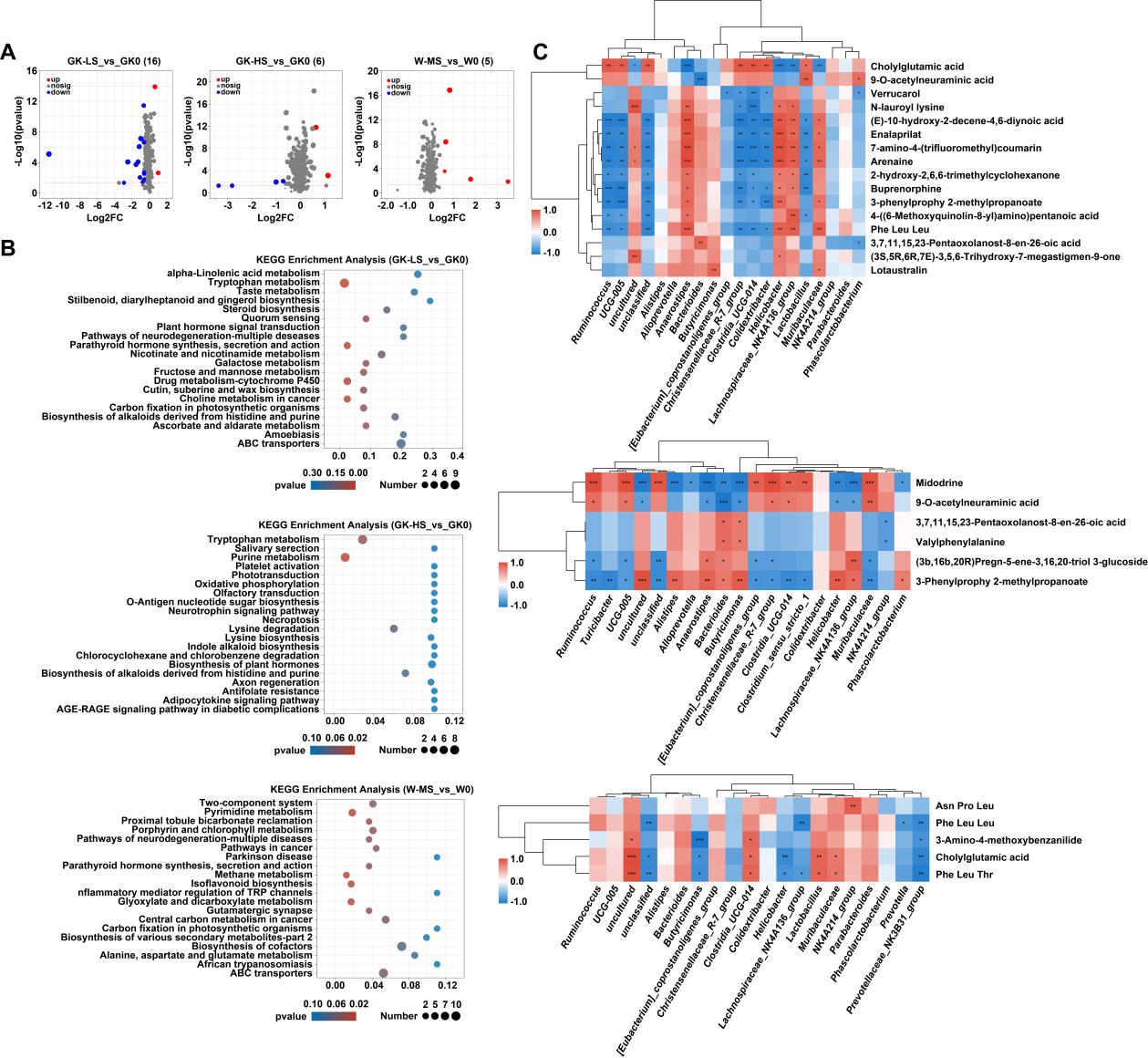


Figure S7 Fecal metabolic patterns in the low (GK4), high (GK8) and medium (W4) dose corn stigma treated groups. (A) Volcano maps of differential metabolites. Significantly upregulated and downregulated genes are shown in red and blue, respectively. Genes with no significant changes (nosig) in transcription are shown in grey. (B) KEGG pathway enrichment analysis. (C) The top is the Pearson correlation of GK4 group, the middle heatmap analysis represent the GK8 group and the bottom represent the W4 group. The red squares indicate positive correlations, whereas the blue squares indicate negative correlations. The metabolite clustering tree is shown on the left. The distance between branches shows the closeness in the expression pattern of metabolites.


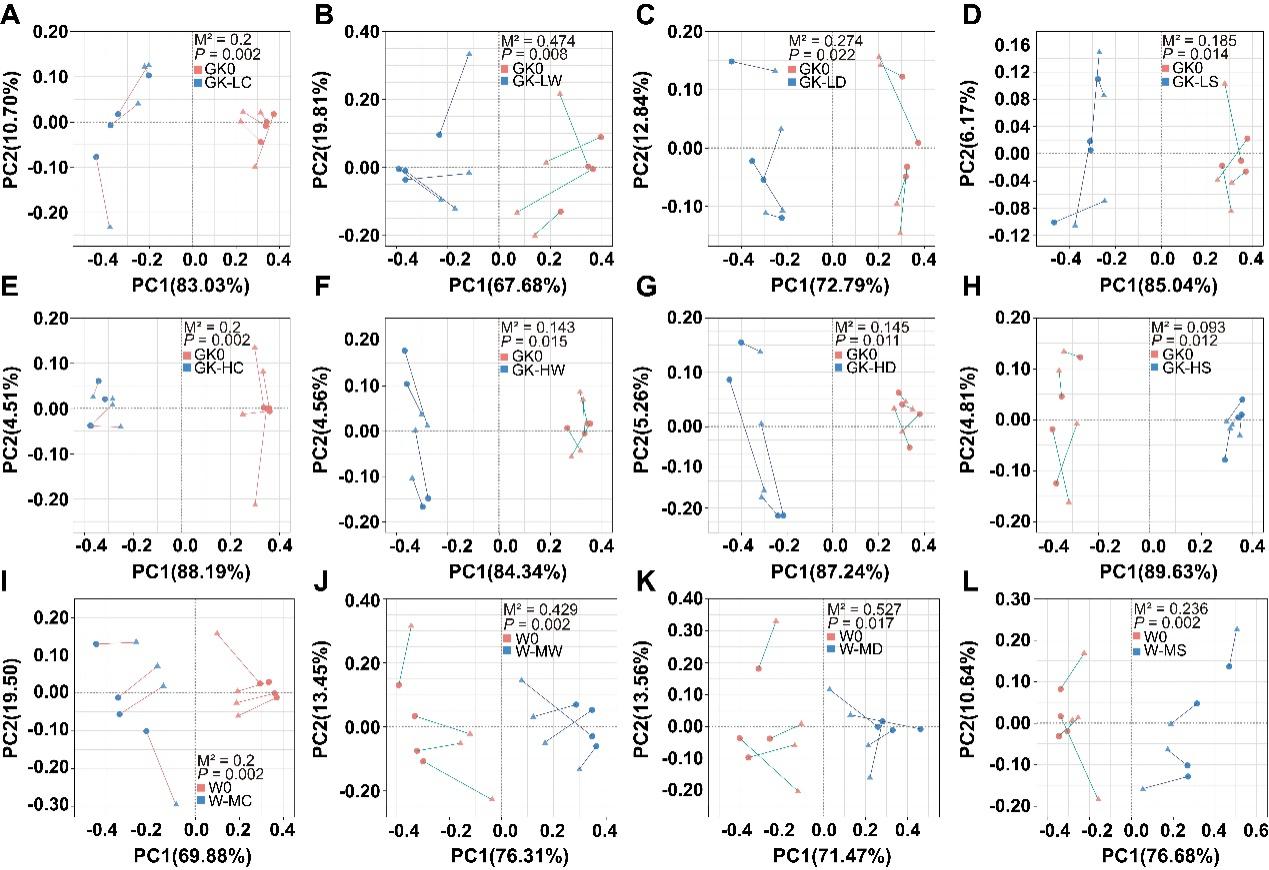


Figure S8 Procrustes analysis for correlations between microbiota and metabolites. The triangles represent metabolites, and the circles indicate16S rRNA gene ASV data. (A), (B), (C), (D) GK groups under low dose dietary drug intervention. (E), (F), (G), (H) GK groups under high dose dietary drug intervention. (I), (J), (K), (L)Wistar groups under medium dose dietary drug intervention.

Table S1 Chemical properties of four dietary drugs.

|  | N/mg | C/mg | S/mg |
| --- | --- | --- | --- |
| Corn starch | 0.11 | 74.64 | 0.34 |
| Activated carbon | 0.11 | 62.13 | 0.77 |
| Wheatgrass | 7.17 | 81.76 | 0.91 |
| Dandelion | 4.60 | 66.93 | 0.72 |
| Corn Stigma | 6.25 | 78.23 | 2.07 |
